# Tools and Resources for Engaging People With Lived and Living Experience and Caregivers in Mental Health and Substance Use Research: Findings From a Survey and Community Consultation Events

**DOI:** 10.1111/hex.70641

**Published:** 2026-03-20

**Authors:** Lisa D. Hawke, Abigail Amartey, Vivien Cappe, Hajar Seiyad, Susan Conway, Joshua Orson, Joshua Dunphy, Sean A. Kidd, Natasha Y. Sheikhan, Nicole Kozloff, Branka Agic, Lina Chiuccariello, Connie Putterman, Melissa Hiebert

**Affiliations:** ^1^ Centre for Addiction and Mental Health Toronto Ontario Canada; ^2^ University of Toronto Toronto Canada

**Keywords:** implementation science, lived experience, mental health, patient engagement, research on research, substance use

## Abstract

**Background:**

There is increasing recognition of the value of engaging people with lived and living experience and caregivers in mental health and substance use health research, in roles such as advisors, collaborators, and co‐researchers. While tools and resources are required to support teams in conducting authentic engagement, it is unclear what is most needed by academic researchers and those with lived/living experience. This study aimed to identify the tools and resources most needed by academic researchers, people with lived/living experience, and caregivers to support engagement and to co‐design a sample of tools.

**Methods:**

We conducted an online survey with 46 people with lived/living experience and caregivers and 46 academic researchers with engagement experience to identify what tools and resources were available and still needed to support engagement in research. We then held two consultations with 55 of the survey participants (38 lived/living experience and caregiver participants, 17 researcher participants). At the consultations, participants discussed the most highly needed tools to be co‐developed. We then conducted a co‐design phase of the project.

**Results:**

While a wide variety of tools and resources are available to people with lived and living experience and academic researchers, many people do not have access to tools and resources that they would consider useful. Participants gave shape to five potential tools and resources. A Lived/Living Experience and Caregiver Working Group then co‐developed three tools—two meeting checklists and a career development tip sheet, which are provided in the Appendices.

**Conclusions:**

A wide variety of tools and resources may be helpful to support engagement. Research teams might consider reviewing the tools and resources that they have, sharing them with the broader engagement community, and seeking those potentially useful items that they would like to have but have not yet accessed. Future work is required to collect the tools available and offer them systematically to the engagement community for more effective lived/living experience and caregiver engagement in mental health and substance use health research.

**Patient and Public Contribution:**

All stages of this study were guided by a Lived/Living Experience and Caregiver Working Group, from grant development to manuscript co‐authorship.

## 1 Background

There is increasing recognition of the value of engaging people with lived and living experience and caregivers in mental health and substance use health research. Engaging them in the research process provides many advantages in terms of producing study findings that are rich in data, have greater authenticity, and are reflective and responsive to real‐world needs [1]. This has led to greater prioritization of engaging people with lived/living experience in research studies as advisors, co‐researchers, co‐producers, or in other similar capacities [1, 2, 3, 4]. People with lived/living experience and caregivers can contribute to all stages of the research, such as identifying emerging research priorities, shaping research questions, identifying aspects of the question with more relevance to the target population, developing study designs, conducting interviews, analyzing data, troubleshooting problems, co‐authoring publications, and expanding knowledge mobilization beyond academic audiences [4, 5]. They can also inform the engagement approach itself. By making engagement a standard practice in research, the research becomes more meaningful, grounded, and accessible to the communities it seeks to serve.

Meta‐research is research conducted on study processes and research methodologies to help improve validity, rigour, reporting, and reproducibility [6]. Meta‐research has the potential for broad‐scale impacts, as its findings improve science by guiding the teams conducting research using the processes and methodologies studied. Conducting lived experience and caregiver engagement in mental health and substance use health research is a meta‐research science in itself. The science of engagement has been influenced by a wide range of original meta‐research studies, reflections, review papers, and meta‐review papers synthesizing findings and recommendations to date [3, 7, 8, 9, 10]. These works have identified many facilitators of authentic engagement, as well as barriers including issues related to communication, team dynamics, researcher readiness, and logistics [1, 11]. Appropriate tools and resources may help research teams implement the facilitators to authentic engagement while mitigating the barriers that often get in the way of optimal engagement practices.

In our team's review of research evidence and practice gaps in the science of mental health and substance use health research engagement, we identified a wide range of research evidence and implementation gaps to be filled [12]. Notably, the meta‐research evidence gaps included conceptualizing lived/living experience, increasing diversity, evaluating engagement, and establishing tools and resources to support engagement. Implementation gaps included a range of broader institutional gaps, such as identifying consistent funding supports and other institutional supports. We also identified gaps in the day‐to‐day practice of engagement, such as understanding relationship building, power dynamics, lived/living experience leadership, and planning processes, while improving available trainings.

In terms of tools and resources needed to support engagement, the literature highlights the need for publications and knowledge translation on engagement, clearer best practice guidelines, procedures, strategies, and, generally, concrete tools, and resources [12, 13, 14]. However, there is a lack of clarity in terms of the types of tools, resources, and supports that are needed. Further research work is required to understand the types of tools, resources, and supports that can help lived/living experience and caregiver partners and academic researchers optimize their engagement experience, and to co‐design such materials together.

## 2 Objective

This project aimed to identify the tools, resources, and supports that people with lived/living experience, caregivers, and academic researchers need to optimize lived/living experience and caregiver engagement in mental health and substance use health research. We sought to understand what these materials should look like through a survey and subsequent consultation events, then to co‐design some relevant tools. The study was designed and conducted in close collaboration with a Lived/Living Experience and Caregiver Working Group.

## 3 Methods

### 3.1 Participants

To be eligible, lived/living experience and caregiver participants had to self‐report having engaged in research projects as advisors, collaborators, co‐researchers, or in analogous roles, in the mental health and/or substance use health research sector. Any of these roles were eligible, as long as the potential participant had experience contributing to research in one of these manners. Researcher participants had to be academic researchers, research trainees, or research staff with experience leading or supporting mental health and/or substance use health research that engages people with lived/living experience and/or caregivers. For both groups, participants had to be 16 years of age or older and residing in Canada.

### 3.2 Recruitment

We recruited *n* = 46 people with lived/living experience and caregivers and *n* = 46 academic researchers. This was a pragmatic survey sample size intended only to collect descriptive information from available parties and gather participants to invite to the consultation events. We recruited by circulating a flyer through our engagement networks, to national and provincial engagement bodies, and via social media (X). The study was featured on the newsletters of multiple engagement‐oriented organizations. We also recruited from three previous Canada‐wide projects on aspects of engagement conducted out of the same institution, from among participants who had provided consent to be contacted about future research.

### 3.3 Procedure

Interested potential participants reached out to the study team by phone, email, or text. A study staff member provided more information on the study, ensured eligibility, and sought written informed consent using the Centre for Addiction and Mental Health (CAMH) e‐consent framework hosted by REDCap [15] on a secure institutional server. After consent, participants received a web link to complete the demographics form and a survey on REDCap. Embedded at the beginning of the REDCap survey was a participant‐friendly, co‐developed video introducing the project and providing instructions on the survey.

Once survey data collection was complete, consultation events were scheduled and all participants were invited. Two consultations were held, one in person at CAMH and one virtually. Members of the Lived/Living Experience and Caregiver Working Group co‐led the events. The purpose of the events was to give shape to the tools, resources and supports identified as most needed, to guide a subsequent co‐design process. Each event began with a short presentation of the survey results. We then used a World Café method [16] to stimulate discussion. We divided participants into multiple breakout groups to discuss one tool or resource, then brought them back for large group discussions There were two breakout sessions during the 90‐min events. The in‐person event had two breakout groups, while the virtual event had three. One tool or resource was discussed in each group, such that each of five tools and resources were discussed twice across the two events. Each breakout session was about 15 min in duration. At the end of the events, participants received a web link or a pencil and paper version of the Public and Patient Engagement Evaluation Tool (PPEET) for the virtual and in‐person events, respectively [17]. In the months after the events, a total of seven co‐design sessions took place. The study was approved by the Research Ethics Board of CAMH.

### 3.4 Measures

The demographic form solicited the basic personal information and engagement backgrounds of participants, including age, gender, ethnicity, and role in research engagement.

The survey queried participants about the tools, resources, and supports they had access to, or still needed, to support authentic engagement. Items were divided into the following categories: community needs (e.g., a regular meeting of lived experience and caregiver partners and/or academic researchers to share experiences), information and training needs (e.g., a list of relevant trainings that are available from various groups across Canada), tool and resource needs (e.g., an engagement planning document to help plan the engagement activities for a project), and institutional needs (e.g., standard operating procedures guiding engagement at the institution). For each question, participants were asked if they had access to the resource, and how useful it is, or how useful it would be to have it, on a five‐point Likert scale (not at all useful to extremely useful). Three open‐ended questions provided the opportunity to offer additional insights. The survey was developed by the full study team, including lived/living and caregiver experience members, through iterative brainstorming and engagement, based on our literature review [12] and our experience conducting engagement. The Lived/Living Experience Working Group discussed the draft items and made revisions together for a relevant survey tool. The survey took about 15 min to complete and included 33 items in total.

The Public and Patient Engagement Evaluation Tool (PPEET) is a tool to evaluate engagement [17]. It was chosen for the consultation events because it includes a version to evaluate a single engagement event and is suitable for completion by people with lived/living experience, caregivers, and academic researchers. This tool consists of 13 questions that are answered on a five‐point Likert scale ranging from strongly disagree (= 1) to strongly agree (= 5), plus open‐ended questions for more information. It contains four subscales: Communication and Supports for Participation, Sharing your Views and Perspectives, Influence of the Engagement Initiative, and Final Thoughts. A total score is derived as a mean score of all items.

### 3.5 Analysis

Demographic data were descriptively summarized for both the survey and the events. Survey data were also descriptively analyzed, including means and standard deviations for each item. Results were analyzed separately based on whether a tool/resource was currently available to the respondent or still needed by them, and separately for lived/living experience and caregiver partners and for academic researchers. We interpreted the results to understand which tools and resources had the highest rate of participants who did not have access to them, yet would consider them useful. Tools meeting these criteria were discussed by the Lived/Living Experience and Caregiver Working Group and five tools and resources were selected by them to take to the consultation events.

For the consultation events, multiple staff members took notes, which were reviewed together and narratively synthesized after the events. Feedback at the consultation events was used to guide our co‐development of the tools/resources.

### 3.6 Co‐Design

After the survey and events, we held seven co‐design sessions to co‐design two tools (Appendix): the meeting checklist and the career development resource. The sessions were attended by two people with lived experience, two caregivers, two research staff members, and one scientist. Activities included reviewing the survey results and event syntheses and using these to select the resources to co‐design based on the data, feasibility within the scope of the project, and their interest. They then brainstormed on the content of the selected resources. A research staff member iteratively drafted the resource between meetings and returned to the meeting to present it to the full team. The Working Group members reviewed each version in detail and provided progressive feedback for improvements. Once the full group reached consensus on the form and format of each resource, they worked on the current manuscript together, then the sessions were concluded.

#### 3.6.1 Lived/Living Experience and Caregiver Engagement

This was a lived/living experience and caregiver‐engaged research project. The project team includes co‐authors with lived and living experience and caregivers who were actively involved in all phases of the research process from grant submission to dissemination. We followed recent Best Practice Guidelines for the engagement of people with lived experience in mental health and substance use health research [5]. We report on engagement following the GRIPP‐2 guideline for reporting on lived experience engagement in research in Table 1 [18].

**Table 1:** Description of lived experience and caregiver engagement on the project.

Domain	Description
Aim	We aimed to conduct this project on engagement, with engagement embedded throughout to maximize relevance.
Methods	We engaged two people with lived/living experience and two caregivers in the project to form a Lived/Living Experience and Caregiver Working Group. Two members were collaborators on the grant application and two were recruited after funding. Some had worked together before and all had previously worked with the project lead on other projects. They were recruited from our previous teams, which facilitated solid working relationships, good experiences working together, and an understanding of each other. We met monthly or bi‐monthly for 15 one‐hour meetings from project launch to manuscript submission. We held icebreakers at the beginning of each meeting to create social engagement. We held regular virtual meetings to review content together, alongside some asynchronous work. All members were compensated according to standard institutional rates. If members missed meetings, we offered one‐on‐one catch‐up sessions if they wished. The project lead was always available to answer questions. The team felt that the lead provided a good environment for comfortable engagement. We navigated disagreement through discussion and compromise.
Study results	The Lived/Living Experience and Caregiver Working Group named the project REACH (*Research Engagement and Collaboration for Health*) and provided revisions to materials such as the pre‐consultation survey, recruitment flyer and demographics form. They guided the team on introductory instructions and an instructional video and advised on recruitment sources. They pilot tested the survey, helped plan the events, reviewed the survey data, selected tools to take to the consultation, co‐facilitated the events, reviewed the field notes together, selected the tools to co‐design, co‐designed the final tools, completed this table together, and co‐authored the manuscript.
Discussion and conclusions	Given the topic area of the project, engagement was an imperative. The Lived/Living Experience and Caregiver Working Group felt that they brought important experience about being engaged in projects and about the tools and resources that they had found and would find useful, which they considered to complement the information provided by research participants. The lived/living experience co‐facilitators seemed to be a strong fit at the events, appearing to create a comfortable consultation space. The co‐design process was a natural continuation of the consultation events and the team agrees that the resulting tools reflect the perspectives of both participants and the Working Group.
Reflections/Critical perspectives	The Working Group members considered the project to be a positive learning experience that increased their sense of competency for future engagement. They felt that it encouraged them to bring other people into research so they can benefit from doing a research project and share knowledge, which they believe builds confidence. They felt that they discovered skills in themselves through the encouragement of the rest of the lived/living experience and caregiver partners. They further felt that engagement helped to break down barriers between people with lived/living experience, caregivers, and academic researchers by having them work together toward a common goal to ultimately improve research processes and outcomes. They felt like they had a substantial impact on the research project. Engagement had similar impacts on academic researchers. They stated that the collaboration specifically built confidence that the research reflected the shared views of all.

## 4 Results

Demographic characteristics are presented in Table 2 for participants who completed the survey and for those who attended the engagement event. The majority of the sample identified as women. There was a range of ages in the group. Just over half of the sample identified as White; several other backgrounds were represented. Research experience was seen across the life stages, with experience in research on children to older adults.

**Table 2:** Demographic characteristics of participants.

Characteristic		Survey (*N* = 92)	Events (*N* = 50)
		*n* (%)	*n* (%)
Age	< 30	30 (32.6)	19 (38.0)
	30–59	34 (37.0)	21 (42.0)
	60+	8 (8.7)	3 (6.0)
	Missing	20 (21.7)	7 (14.0)
Gender	Man	12 (13.0)	7 (14.0)
	Woman	71 (77.2)	39 (78.0)
	Transgender, gender non‐binary	8 (8.7)	4 (8.0)
	Missing	1 (1.1)	0 (0.0)
Racial/ethnic background	White	47 (51.1)	23 (46.0)
	South Asian	16 (17.4)	13 (26.0)
	East/Southeast Asian	8 (8.7)	4 (8.0)
	Black	2 (2.2)	2 (4.0)
	Middle Eastern	6 (6.5)	5 (10.0)
	Multiple heritages	7 (7.6)	1 (2.0)
	Another heritage	3 (3.3)	1 (2.0)
	Missing/Don't know	3 (3.3)	1 (2.0)
Born in Canada	Yes	66 (71.7)	35 (70.0)
	No	26 (28.3)	15 (30.0)
Highest education	High school or less	4 (4.3)	1 (2.0)
	Some post‐secondary	7 (7.6)	6 (12.0)
	Post‐secondary degree, diploma, certificate	28 (30.4)	19 (38.0)
	Post‐graduate degree	49 (53.3)	22 (44.0)
	Missing	4 (4.3)	2 (4.0)
Role	Person with lived/living experience (only)	28 (30.4)	22 (44.0)
	Caregiver (only)	6 (6.5)	3 (6.0)
	Both person with lived/living experience and caregiver	10 (10.9)	8 (16.0)
	Researcher	46 (50.0)	16 (32.0)
	Missing^a^	2 (2.2)	1 (2.0)
Research background^b^	Child‐oriented research	14 (15.2)	8 (16.0)
	Youth‐oriented research	61 (66.3)	34 (68.0)
	Adult‐oriented research	60 (65.2)	30 (60.0)
	Geriatrics‐oriented research	7 (7.6)	6 (12.0)
Area of Canada	Western Canada	20 (21.7)	8 (16.0)
	Central Canada	67 (72.8)	40 (80.0)
	Eastern Canada	4 (4.3)	1 (2.0)
	Northern Canada	1 (1.1)	1 (2.0)

^a^ Missing values were individuals from the lived experience/caregiver sample who did not indicate whether they had personal or caregiver lived experience.

^b^ Sums exceed 100% due to the option to check all that apply.

Survey results are presented in Table 3. A majority of lived/living experience and caregiver respondents had access to tools like newsletter updates, regular meetings, free virtual/in‐person trainings, and institutional leads who support engagement—all of which were considered very useful or higher. Among researcher participants, a majority had access to free virtual/in‐person trainings, information sheets on practical key components, sample stories or articles describing engagement, and institutional leads that support engagement, which were considered somewhat useful or higher. Across samples, some of the tools and resources that were accessible to the fewest participants were tip sheets on career development initiatives, guidelines on reporting on engagement and publishing together, a lived experience bill of rights, ethics board materials, templates to help people with lived/living experience and caregivers develop goals, specific trainings, and an online discussion board or forum, with varying usefulness ratings.

**Table 3:** Results of the pre‐consultation survey on engagement support needs, categorized by access to resource and perceived usefulness for each participant group.

	People with lived/living experience and caregivers (*n* = 46)			Academic researchers (*n* = 46)		
Items	*n* (%) with access	With access	No access	*n* (%) with access	With access	No access
		Usefulness M (SD)	Usefulness M (SD)		Usefulness M (SD)	Usefulness M (SD)
**Community needs**						
1. Regular newsletter updates	26 (57.8)	4.1 (0.9)	4.1 (0.9)	18 (39.1)	3.7 (0.8)	3.8 (1.0)
2. A regular meeting of people with lived/living experience, caregivers, and/or researchers	24 (53.3)	4.2 (0.8)	3.5 (1.1)	17 (37.8)	3.9 (0.9)	3.9 (1.0)
3. An online discussion board or forum	12 (26.7)	4.1 (1.0)	3.4 (1.3)	6 (13.3)	3.7 (1.0)	3.4 (1.2)
4. Shadowing, mentorship, or a buddy system	12 (26.7)	4.3 (1.1)	3.9 (1.1)	10 (22.2)	4.5 (0.7)	3.7 (1.1)
**Training needs**						
5. A list of relevant engagement trainings^a^	14 (31.1)	4.0 (1.0)	4.1 (0.7)	13 (28.3)	3.5 (1.1)	4.2 (0.9)
6. Free training: virtual or in‐person	26 (57.8)	4.0 (0.9)	4.2 (0.8)	27 (58.7)	3.7 (1.1)	3.7 (1.3)
7. Free training online modules	15 (33.3)	3.7 (1.3)	3.6 (1.2)	16 (34.8)	3.2 (1.4)	3.5 (1.2)
8. Training on interpersonal aspects^a^	12 (27.3)	4.0 (1.0)	3.6 (1.2)	9 (19.6)	4.0 (0.7)	4.1 (1.1)
9. Training on how to build active teams	18 (40.0)	4.0 (0.9)	3.8 (1.2)	8 (17.4)	4.1 (0.6)	3.9 (1.1)
**Tools and resources**						
10. Information sheets on practical key components	20 (44.4)	4.0 (0.9)	3.9 (0.9)	27 (58.7)	4.1 (0.8)	4.0 (1.0)
11. Information on engagement in funding applications	16 (35.6)	3.9 (1.0)	3.2 (1.1)	13 (28.3)	3.8 (1.0)	3.7 (1.1)
12. Information about project‐specific guidance documents	17 (37.8)	3.8 (1.2)	3.4 (0.9)	20 (43.5)	3.9 (0.9)	3.9 (0.9)
13. An information sheet about research, from start to finish	17 (37.8)	4.4 (0.9)	4.0 (0.8)	18 (39.1)	3.7 (0.8)	4.1 (1.0)
14. An engagement startup package^a^	16 (35.6)	4.4 (1.0)	4.0 (0.9)	9 (20.0)	3.9 (1.5)	4.4 (0.9)
15. Sample stories or articles describing engaged research projects	15 (33.3)	4.3 (0.8)	3.5 (1.2)	28 (60.9)	3.6 (1.2)	3.4 (1.3)
16. An engagement planning document	19 (42.2)	4.0 (1.0)	3.6 (0.9)	14 (30.4)	3.9 (1.0)	3.9 (1.0)
17. Templates to help develop advisors' goals	12 (26.7)	4.0 (1.0)	3.6 (1.0)	9 (19.6)	4.1 (0.8)	3.6 (1.1)
18. A meeting preparation checklist^a^	11 (24.4)	4.2 (1.1)	3.8 (1.0)	13 (28.3)	4.2 (0.8)	3.9 (1.0)
19. Glossaries with lay‐friendly terminology	13 (28.9)	4.6 (0.7)	3.9 (1.0)	12 (26.1)	4.1 (1.1)	3.8 (1.3)
20. Guidelines on writing and publishing together	10 (22.2)	4.2 (0.9)	4.2 (0.8)	13 (28.3)	4.1 (0.8)	4.1 (1.1)
21. Guidelines on how to report on engagement	9 (20.0)	4.1 (0.9)	3.9 (1.0)	13 (28.3)	4.1 (0.8)	4.2 (1.0)
22. Guidelines and tools on how to evaluate	15 (33.3)	3.9 (1.0)	4.1 (0.9)	21 (45.7)	3.9 (0.9)	4.5 (0.7)
23. Information sheet on how to recruit lived/living experience and caregiver partners	13 (28.9)	4.1 (1.0)	3.7 (0.8)	15 (32.6)	4.1 (1.1)	3.7 (1.0)
24. Tip sheet on providing people with lived/living experience and caregivers with career development initiatives^a^	8 (17.8)	4.1 (1.2)	4.2 (1.2)	2 (4.3)	5.0 (0.0)	3.6 (1.2)
**Institutional needs**						
25. Standard operating procedures	11 (25.0)	3.8 (1.3)	3.5 (0.9)	18 (39.1)	4.0 (0.8)	3.9 (1.2)
26. A bill of rights for engagement	9 (20.0)	4.1 (0.9)	3.9 (1.1)	6 (13.0)	3.8 (0.8)	3.5 (1.3)
27. Materials to raise awareness of engagement among Research Ethics Boards	9 (20.0)	3.8 (1.2)	3.8 (1.0)	5 (10.9)	3.4 (0.5)	3.7 (1.2)
28. Professional engagement roles for staff	19 (42.2)	4.1 (1.0)	3.5 (1.1)	20 (43.5)	4.6 (0.6)	3.4 (1.1)
29. Institutional leads who prioritize engagement	26 (57.8)	4.2 (0.9)	4.4 (0.9)	30 (66.7)	4.6 (0.7)	4.0 (1.1)

*Note:* ‘With access’ refers to participants who responded that they had access to the tool or resource in question, while ‘no access’ refers to participants who did not have access to it. Note that the percentages are calculated based on the number of participants who responded to each item; denominators vary slightly due to item‐level missing data. Usefulness ratings are on a 1‐5 Likert scale, where 5 represents ‘extremely useful.

^a^ These items were taken forward to the consultation events.

The next stage was two consultation events. The in‐person event was attended by *n* = 12 participants, and the virtual event by *n* = 43 attendees, for a total of *n* = 55 attendees (demographics missing for *n* = 5). Among the 55 attendees, there were 38 lived/living experience and caregiver participants and 17 researcher participants. Based on the sum of the findings in Table 3, the team decided to bring five tools and resources to the consultation events, with a primary focus on items that were available to the fewest participants but rated as highly useful. The five items are listed below, with a brief synopsis of the feedback collected at the consultation events.

### 4.1 A List of Relevant Engagement Trainings That Are Available Across Canada

Attendees shared about the training activities that they had accessed and those that they had heard of within their networks. A variety of trainings were identified, targeting people with lived/living experience, caregivers, and academic researchers, across a wide range of recognized organizations, including funders, patient‐oriented research support units, universities, and research organizations. Trainings addressed core engagement principles, as well as important related knowledge bases and skill sets such as knowledge translation, culturally informed care, Indigenous knowledge, and anti‐oppression training. A variety of other complementary resources were also shared, such as toolkits, workbooks, and websites. Attendees felt that these trainings should be available to all and should be co‐produced with lived/living experience and caregiver partners. They described these resources as a useful standardized print document to be shared across a wide variety of accessible methods such as social media and email. Participants felt that, optimally, any list of engagement trainings should include a vetting process whereby the trainings are evaluated and scored, to help users make an informed decision about which trainings to choose.

### 4.2 Training Specifically on Interpersonal Aspects Like Developing Strong Group Dynamics, Relationships, and Rapport

Attendees shared a few engagement trainings that address components of the interpersonal aspects of engagement, including some of the trainings listed above. They thought it was important to train academic researchers and people with lived/living experience and caregivers on issues such as diversity, cultural sensitivity, life skills, mental health, power dynamics, complaints processes, avoiding jargon, and lay language. They felt that trainings should be tailored to specific populations rather than generic in nature. They preferred live training opportunities over resource documents for this type of information, although they felt that resource documents could be made available online and hosted on a credible institution's website. Attendees pointed to the importance of compensating lived/living experience and caregiver partners for their training time, providing them with opportunities to co‐develop or co‐facilitate the training, and be inclusive of more marginalized communities.

### 4.3 A Startup Package to Help Onboard New People With Lived/Living Experience and Caregivers to Research Projects

Attendees shared a variety of potential components of a start‐up package, such as an overview of engagement, lived experience‐friendly research glossaries, a list of available trainings, information or questionnaires to help identify roles and skills, information on research methodologies, and contact information. They emphasized the need for plain language descriptions of engagement, the study and any related grant application, the study team, and engagement roles. Being clear and transparent about any information sharing and the engagement plan was important to them, as was transparency about any information that would be shared about them. They thought that this package could be offered in the form of a meeting or webinar, or alternatively in document format with visual materials or a mobile application. They emphasized the importance of diversity and accessibility regardless of the format chosen.

### 4.4 Tip Sheet on Providing People With Lived/Living Experience and Caregivers With Career Development Initiatives to Further Their Growth Within Research and Within the Institution

Attendees expressed that lived/living experience and caregiver partners can be supported in developing their careers by helping develop their interests and skills, setting realistic expectations, allowing them to take an active role throughout the project, and recognizing their achievements. They felt that a tip sheet on career development opportunities might address issues like co‐authorship and conference attendance opportunities; opportunities to help create posters, slide decks and reports to develop their document skills; and making full or part‐time roles available to support studies. They emphasized that a fair, safe, and inclusive environment and mentorship opportunities would help them develop their skills. They pointed out that some lived/living experience and caregiver partners might appreciate information about what a career in research looks like. Importantly, they noted that not all people with lived/living experience are interested in career development opportunities and this should therefore be an optional resource. They felt that this would be a useful document as a handout, possibly with infographics or a decision tree, potentially on a website. This resource was co‐designed in Stage 3 (Appendix A).

### 4.5 A Checklist to Help Academic Researchers and Lived/Living Experience Team Members Prepare for Effective Research Engagement Meetings

The essential content for this resource included statements about timeliness and best practices for engagement, meeting expectations, and a note about a terms of reference document for the group. Further content included a note about allowing time at the end of meetings for questions and specific introductory meeting items such as time for the researcher to explain why they are doing this work. Attendees felt that the checklist should include instructions to share meeting agendas before meetings, hold pre‐briefs if possible, and have icebreakers at the beginning of the meeting. They also suggested including an overview of ‘housekeeping’ items, providing time to build relationships and rapport, and providing clear information about compensation. Attendees felt that the checklist should include instructions for academic researchers to meet individually with people with lived/living experience and caregivers before starting a meeting series to prepare them and that they should be specifically attentive to any potentially stigmatizing language. They felt that this checklist could be an online or print resource. We co‐designed this resource in Stage 3, in the form of a set of two checklists (one for lived/living experience and caregiver partners, one for academic researchers; Appendices B and C).

#### 4.5.1 Evaluation

PPEETs were completed by *n* = 47 event attendees (85%). Across the scale, the average total score was 4.2 (SD = 0.47, which equates to just over the ‘Agree’ anchor on the Likert scale). For the subscales, the average score for *Communication and Supports for Participation* was M = 4.3 (SD = 0.53). For *Sharing your Views and Perspectives*, we averaged 4.3 (SD = 0.60). For Impacts and *Influence of the Engagement Initiative*, the average was 4.2 (SD = 0.66). Lastly, for the *Final Thoughts* section the average was 4.2 (SD = 0.69). As a whole, these ratings suggest that the consultation events were worthwhile and a positive experience for most attendees. Open‐ended results reflected that participants were largely satisfied with the event (planning, environment, facilitation, discussions), although they would have preferred a 2 h consultation instead of 90 min.

## 5 Discussion

This study included an online survey and two consultation events aiming to understand what tools and resources lived/living experience and caregiver partners and engaged academic researchers need to optimize lived/living experience and caregiver engagement in mental health and substance use health research. The survey suggested that there is a perceived need for a wide range of tools and resources, while the consultation events explored content that could be included in five high‐priority tools and resources. Through seven co‐design sessions, a Lived/Living Experience and Caregiver Working Group worked together with academic researchers to develop three tools, which are provided. This project provides information for lived/living experience, caregiver, and research teams working on the science of engagement, to help guide their tool development initiatives.

It is important to note that all of the listed tools and resources were available to at least some participants. This finding illustrates that there are many Canadian teams doing engagement and likely developing their own tools to support their practice. However, most tools and resources were available to only a minority of participants, suggesting that useful items are not being shared across engagement spaces. Many more research teams could benefit if dissemination were more widespread. It is well known that effective information dissemination is a research gap [19]. This extends to the science of lived/living experience and caregiver engagement. While collecting the tools and resources was beyond the scope of the current project, a future project might include a comprehensive environmental scan of tools and resources, following established environmental scan methodology [20], together with the development of a web portal sharing them broadly to the engagement community to minimize duplication. Challenges with the resource recommendations include identifying resources to pay people with lived experience and caregivers for attending to trainings and keeping a comprehensive list of trainings evaluated and up to date.

The tool that the fewest participants had access to across both samples was the tip sheet on providing lived/living experience and caregiver partners with career development initiatives, a tool that we went on to co‐design (Appendix A). Members of our co‐design team felt that this should extend beyond the development of research careers, to include career development as a whole, and that it should be a shared resource and responsibility across people with lived experiences, caregivers, and researchers. While this is broad in scope, this was the preference expressed by participants. The career development of lived/living experience and caregiver partners may not always be top of mind for academic researchers, but investing in their development and leadership skills can produce very positive consequences for the individuals and the field as a whole. While the literature nods to career development issues regarding engagement for academic researchers [21, 22], training and skill development opportunities have also been called for in engagement [23, 24], which can be expanded to encompass career development skills and opportunities. It is important to explore the needs of the people with lived/living experience and caregivers to understand what would be meaningful for them in order to create a positive engagement experience [5]. Although not all are seeking career development opportunities, some would find this helpful. Our co‐developed tip sheet can serve to guide discussions between academic researchers and lived/living experience partners. Career development opportunities might make the engagement more meaningful to the people engaged, while providing them with opportunities to make a broader range of contributions to the research. While developed in a mental health and substance use health context, this tip sheet might find relevance in broader health engagement contexts.

The second set of resources that we co‐designed was a set of two checklists to help people with lived/living experience, caregivers, and academic researchers prepare for meetings (Appendices B and C). Our Working Group considered this to be a high priority to co‐design, as a broadly applicable resource. It should be noted that similar guidance for academic researchers is available online, as stand‐alone checklists or components of larger informational resources [25, 26, 27]. The content is similar, yet somewhat customized based on the consultation and co‐design sessions. Our tool set also includes a parallel checklist to help lived/living experience and caregiver partners prepare for meetings, which is rarer in the literature. Academic researchers, lived/living experience and caregiver partners, and trainees are encouraged to access any of these tools for their teams, as they may be relevant to initial onboarding trainings and ongoing support, potentially beyond mental health and substance use health contexts.

One key resource that was particularly highly rated was having institutional leads who prioritize engagement. Having strong institutional support is an important facilitator of engagement [1]. Indeed, the implementation science literature frequently discusses the importance of having a ‘champion’ to implement practices in the healthcare sector [28]. Fortunately, many participants in our sample had access to this, although still a third of researcher participants and over 40% of those with lived/living experience and caregivers did not. In order to embed engagement effectively within a research institution, it is essential that the institutional leads believe in its value and be willing to support and fund their scientists in working in this manner. Institutions might consider embedding engagement as a value in their strategic plans, training research leadership on its value, and actively supporting the scientists who are working in this manner. Embedding people with lived/living experience and caregivers at the leadership level could be a further helpful resource and facilitator of strong engagement [29, 30]. This would be a key resource that would help expand engagement within and across institutions.

Additional tools that were highly prioritized were guidelines on how to write manuscripts with people with lived/living experience and caregivers and how to report on engagement in manuscripts. While the high ratings suggested that we could have discussed these at the consultation events, we did not. This was because members of our team have recently developed such guidance specific to mental health and substance use health, which complement pre‐existing reporting guidelines for health as a whole [18, 31]. We provided our team's tools to event attendees, as greater dissemination is clearly required. Future work is required to further conceptually validate the utility of these tools and disseminate them more widely.

## 6 Strengths and Limitations

We engaged with lived/living experience and caregiver partners at all stages of the project. Participants reflected the range of research target populations across the lifespan. However, a majority of participants identified as women and Canadian born. Although 10 of Canada's 13 provinces/territories were represented, a majority were from Central Canada. We were unable to bring all of the tools and resources to the consultation event and were only able to co‐design a few tools. Future projects can serve to co‐develop additional tools to support engagement and public dissemination. Self‐selection to participate may have introduced an additional bias, as the sample may consist of the people most heavily engaged in engagement spaces rather than newcomers to the engagement space. More than half of the people with lived/living experience and caregivers engaged had a post‐graduate degree, making it a highly educated sample. It should be noted that the co‐produced tools have not been pilot tested or validated in a research setting. They may be subject to ongoing development.

## 7 Conclusions

This project provides an overview of the types of tools and resources that can support engagement, along with their actual and perceived usefulness to lived/living experience and caregiver partners and academic researchers. Research teams might consider reviewing the tools and resources that they have, sharing them with the broader engagement community, and seeking those useful items that they would like to have but have not yet accessed. Reviewing them in an engagement setting, with the people with lived/living experience and caregivers on the team, will help ensure that they are appropriate to any given research team. Future lived experience‐engaged environmental scan work is required to collect the tools available, create a synthesis of them, and offer them systematically to the engagement community for more effective engagement in mental health and substance use research.

## Author Contributions

**Lisa D. Hawke:** conceptualization; investigation; funding acquisition; writing – original draft; methodology; supervision; resources; project administration; formal analysis. **Abigail Amartey:** conceptualization; investigation; writing – review and editing; methodology; formal analysis; project administration. **Vivien Cappe:** conceptualization; investigation; methodology; writing – review and editing. **Susan Conway:** conceptualization; investigation; methodology; writing – review and editing. **Joshua Orson:** conceptualization; methodology; writing – review and editing; investigation. **Joshua Dunphy:** conceptualization; investigation; methodology; writing – review and editing. **Sean A. Kidd:** conceptualization; investigation; writing – review and editing; methodology. **Branka Agic:** conceptualization; investigation; methodology; writing – review and editing. **Lina Chiuccariello:** conceptualization; investigation; methodology; writing – review and editing. **Connie Putterman:** conceptualization; investigation; methodology; writing – review and editing. **Melissa Hiebert:** conceptualization; investigation; methodology; writing – reveiw and editing.

## Ethics Statement

This study was approved by the Research Ethics Board of the Centre for Addiction and Mental Health (#2024‐178). All participants provided electronic informed consent to participate.

## Consent

All participants provided informed consent to the use of their data for research purposes and publication.

## Conflicts of Interest

The authors declare no conflicts of interest.

## Supporting information

Appendix A ‐ Career Development Tip sheet.

Appendix B ‐ Engagement meeting checklist ‐ Lived experience.

Appendix C ‐ Engagement meeting checklist ‐ Researchers.

## Data Availability

The data underlying this manuscript are governed by the Research Ethics Board of the Centre for Addiction and Mental Health and may be available upon request with Research Ethics Board approval.
