# Supplementary material for: Tools and Resources for Engaging People With Lived and Living Experience and Caregivers in Mental Health and Substance Use Research: Findings From a Survey and Community Consultation Events
Source: Health Expect. 2026 Mar 20;29(2):e70641. doi: 10.1111/hex.70641 (PMC13080891; doi:10.1111/hex.70641)
Supplement: Supplementary file 1 — Appendix A ‐ Career Development Tip sheet. [file HEX-29-e70641-s003.pdf]

# CAREER DEVELOPMENT
# Tip Sheet

***For research teams including people with lived and living experience and caregivers engaged in research***

*This resource is to support the development of any kind of career or personal skills. Many skills that people with lived/living experience and caregivers can develop in research contexts are transferable to other careers. This should be kept in mind when discussing career development.*

## GETTING STARTED

- Open a **conversation** about career and personal development, including research careers — note that not all people with lived/living experience and caregivers are interested in these supports.
- Share **examples, success stories,** and **networks** to make career pathways and institutional development feel possible.

## FOR RESEARCHERS: HOW TO SUPPORT PEOPLE WITH LIVED/ LIVING EXPERIENCE AND CAREGIVER DEVELOPMENT

- **Identify areas** of interest and skill development with people with lived/living experience and caregivers and create meaningful opportunities in those areas.
- Recognize current and past **transferable skills** to help people with lived/living experience and caregivers obtain jobs and advance in them (e.g., group facilitation, document writing and review, recruitment support, working with diverse groups, public speaking, computer literacy skills, etc.).
- Recognize and leverage **less tangible skills** (e.g., strategic thinking, peer leadership).
- Provide **guidance and mentorship**, particularly for those who are early-career or youth.
- Involve people with lived/living experience and caregivers **throughout the research process** from initial planning to publication and knowledge translation.
- Offer **safe, inclusive environments** with fair pay, networking access, and decision-making opportunities.

- **Avoid bias** towards people with lived/living experience and caregivers based on differing levels of experience (e.g., those with academic, previous research, or health system experience).
- Where feasible, explore full and part-time **employment** on research teams. When projects expire, consider bridging opportunities or other opportunities within the institution.
- Create a structure where people with lived/living experience and caregivers can **mentor** others entering into research, if all are interested.

## FOR PEOPLE WITH LIVED/LIVING EXPERIENCE AND CAREGIVERS: HOW TO GROW WITHIN AN ORGANIZATION

- Have **open discussions** about mutual and realistic expectations for your role.
- Identify **existing skills and abilities** and find ways to apply them in research projects.
- Seek out **peer mentorship** opportunities.
- Where possible, explore **employment or additional advisory opportunities** on research teams.

## A FEW AREAS FOR CONCRETE SKILL DEVELOPMENT

- **Poster, slide deck,** and **report creation** to build practical skills.
- **Meeting facilitation**, **collaboration**, and **teamwork**.
- Attendance at **conferences, workshops,** and **training sessions**.
- **Co-authorship** when contributions meet authorship guidelines.

*This tips sheet was developed collaboratively among researchers and people with lived/living experience and caregivers based on their experience doing engagement together.*

**Suggested citation**: Lisa D. Hawke, Abigail Amartey, Jingyi Hou, Vivien Cappe, Hajar Seiyad, Susan Conway, Joshua Orson (2026). Career development tips sheet for research teams including people with lived and living experience and caregivers engaged in research. Centre for Addiction and Mental Health, Toronto, Canada
