# Supplementary material for: Tools and Resources for Engaging People With Lived and Living Experience and Caregivers in Mental Health and Substance Use Research: Findings From a Survey and Community Consultation Events
Source: Health Expect. 2026 Mar 20;29(2):e70641. doi: 10.1111/hex.70641 (PMC13080891; doi:10.1111/hex.70641)
Supplement: Supplementary file 2 — Appendix B ‐ Engagement meeting checklist ‐ Lived experience. [file HEX-29-e70641-s002.pdf]

ENGAGEMENT MEETING Checklist

For people with lived/living experience and caregivers

***Reminder**: Your expertise is valuable, important, and essential to these conversations.*

BEFORE THE MEETING

- [ ] Attend any pre-meeting that are part of the onboarding process, as they will give you the opportunity to ask questions and get clarity on the project.
    - If there is no pre-meeting, consider asking any questions by email/phone, or asking whether there are any engagement specialists who can meet with you for onboarding.
- [ ] Review the meeting agenda and materials if provided. If you wish to provide feedback in advance, consider sending your thoughts by email.
- [ ] Prepare anything you have been asked to prepare for the meeting (e.g., introduction, icebreaker).
- [ ] Prepare questions or feedback related to the project, materials, and meetings.
- [ ] Clarify the compensation options and process if necessary.
    - Choose what works best for you and ask for any alternatives you may prefer (e.g., cash, e-transfer, gift card).

DURING THE MEETING

- [ ] Listen actively to the content of the meeting.
- [ ] Bring forward ideas, questions, and feedback.
- [ ] Engage with the meeting content and the team, in whatever way feels comfortable.
- [ ] Be mindful of the agenda; ensure that the speaking time is shared among attendees.
- [ ] Ask for clarification on any terms, decisions, or expectations.
- [ ] Use any designated Q&A time to provide additional input if you have any.

AFTER THE MEETING

- [ ] Review any meeting notes and/or follow-up items that the team provides.
- [ ] Track your hours for compensation and submit when appropriate.
- [ ] Reflect on your experience and provide feedback if you wish.
    - If you were struggling to express yourself or you had additional comments, consider asking research staff for a debrief meeting or providing feedback in an email.
- [ ] Complete any post-meeting follow-up or evaluation if received.

This checklist was developed collaboratively among researchers, people with lived/living experience, and caregivers based on their experience doing engagement together.

Suggested citation: Lisa D. Hawke, Jingyi Hou, Abigail Amartey, Vivien Cappe, Hajar Seiyad, Susan Conway, Joshua Orson (2026). Engagement meeting checklist for people with lived/living experience and caregivers. Centre for Addiction and Mental Health, Toronto, Canada.
