# Supplementary material for: Tools and Resources for Engaging People With Lived and Living Experience and Caregivers in Mental Health and Substance Use Research: Findings From a Survey and Community Consultation Events
Source: Health Expect. 2026 Mar 20;29(2):e70641. doi: 10.1111/hex.70641 (PMC13080891; doi:10.1111/hex.70641)
Supplement: Supplementary file 3 — Appendix C ‐ Engagement meeting checklist ‐ Researchers. [file HEX-29-e70641-s001.pdf]

ENGAGEMENT MEETING Checklist

For researchers/research staff

BEFORE MEETINGS

- [ ] Prepare a meeting agenda (with a summary from the previous meeting if applicable) and send it with a meeting reminder to all attendees before each meeting (e.g., the day before, the morning of).
- [ ] Provide any documents you would like attendees to review and refine, ideally at least 3 days in advance. Indicate sections where feedback from people with lived/living experience and caregivers is most needed.
- [ ] Set clear meeting expectations for each meeting, including:
  - Meeting purpose, structure, and time commitments
  - Your engagement goals and expected outcomes, while staying open to novel feedback
  - What material can and cannot be changed (with reasons)
  - Accessibility accommodations (e.g., technology, supports)
- [ ] Prepare an ice-breaker or check-in activity for each meeting.
- [ ] Reflect on power, language, and inclusion:
  - Be aware of any stigmatizing terms, bias, or assumptions.
  - Be mindful of tone and posture; acknowledge power dynamics.
  - Commit to inclusive and empowering communication.
- [ ] Prepare a relaxed and welcoming setting for in-person meetings:
  - Some researchers and people with lived/living experience and caregivers might appreciate certain accommodations, while others might not. Ask whether some of these would be appropriate to them: casual seating, snacks, art supplies, fidget toys, etc.

This checklist was developed collaboratively among researchers, people with lived/living experience, and caregivers based on their experience doing engagement together.

Suggested citation: Lisa D. Hawke, Jingyi Hou, Abigail Amartey, Vivien Cappe, Hajar Seiyad, Susan Conway, Joshua Orson (2026). Engagement meeting checklist for researchers/research staff. Centre for Addiction and Mental Health, Toronto, Canada

DURING MEETINGS

- [ ] Begin and end meetings promptly to respect everyone's time.
- [ ] Begin the meeting with a brief icebreaker activity or check-in, depending on group preference.
- [ ] Open with a reminder of the project status.
- [ ] Ensure clarity and inclusivity in communication:
  - Revisit decisions as needed.
  - Use non-stigmatizing and accessible language.
  - Use clear and everyday language, and explain technical terms when needed.
  - Show appreciation consistently.
- [ ] Regularly provide constructive feedback to people with lived/living experience and caregivers about their contributions.
  - Provide updates on how their feedback in previous sessions has been used or incorporated into the project.
- [ ] Include structured opportunities for input and leadership:
  - Be mindful that either the full meeting or a portion of the meeting should be about collecting perspectives from people with lived/living experience and caregivers.
  - Be flexible, recognizing that all people are different.
  - Facilitate the conversation in a way that encourages input from all in a variety of ways (e.g., speaking up, commenting in the chat, email).
  - Welcome and consider all feedback, even if it falls outside your planned goals.
- [ ] Stay on topic and redirect the conversation respectfully when needed.
- [ ] Make time for check-ins with people with lived/living experience and caregivers, depending on the feel of the group.
- [ ] Include a mid-meeting break for meetings longer than 1 hour.
- [ ] Dedicate time at the end of the meeting for questions and reflections.
- [ ] Confirm when you'll connect with them about next steps.

AFTER MEETINGS

- [ ] Process payments for people with lived/living experience and caregivers promptly and let them know when they should expect to receive their funds.
- [ ] Send out action items, decisions, and a meeting summary, along with a thank-you message, thanking people with lived/living experience and caregivers for their time and insights.
- [ ] Send an expected schedule for the upcoming meeting(s).
- [ ] Send post-meeting follow-up materials or evaluations as needed.
- [ ] If applicable, return to the group (or send an update) to explain how any remaining feedback was addressed in final project outcomes. If it was not, state why not.
- [ ] If you have evaluated engagement, apply any relevant process changes.

INITIAL MEETING CONSIDERATIONS

Before the initial meeting

- [ ] Prepare a concise and easy-to-read project overview; keep it within one to two pages. Send out at least 3 days before the initial meeting.
- [ ] Have a Terms of Reference document to share with the group, or develop one together in the first meeting, including (but not limited to):
    - Project objectives and engagement goals
    - Roles, responsibilities, and staff contact information
    - Compensation details (e.g., method of payment, expenses, tracking & submitting hours)
    - Methods for sharing project info (e.g., Google Docs, email, cloud, printed documents)
    - Accessibility considerations
    - Decision-making process
    - Communication preferences (e.g., email, phone, etc.)
    - Complaint/conflict resolution process, including how concerns can be raised and addressed
    - Other important details
- [ ] Consider establishing accountability mechanisms, such as a bill of rights, shared commitments, or an accommodation plan.
- [ ] Schedule one-on-one meetings with each person with lived/living experience and/or caregiver to understand their goals, strengths, areas for growth, and logistical needs (e.g., compensation).
- [ ] Check for dietary restrictions for in-person meetings when applicable.

During the initial meeting

- [ ] Invest in building relationships and rapport before starting the work.
- [ ] Open with a short welcome round, including:
    - Brief introductions
    - What people with lived/living experience and caregivers hope to achieve; adapt roles or approach if needed.
- [ ] Provide a reminder of roles and expectations.
- [ ] Share contact info for yourself and any available engagement specialists so they can send further feedback.
- [ ] Assess advisor availability and monitor changes over time.
- [ ] Discuss the terms of reference and shared commitments and revise them together.
- [ ] Share your motivations for the work (e.g., why this research matters to you, why you value engaging people with lived/living experience and caregivers)
- [ ] Provide a timeline for the project and its engagement components, including important milestones.
- [ ] Review the meeting summary together and answer any questions.
